# Potential Treatment of Dermatophyte *Trichophyton rubrum* in Rat Model Using Topical Green Biosynthesized Silver Nanoparticles with *Achillea santolina* Extract

**DOI:** 10.3390/molecules28041536

**Published:** 2023-02-05

**Authors:** Basem M. Abdallah, Peramaiyan Rajendran, Enas M. Ali

**Affiliations:** 1 Department of Biological Sciences, College of Science, King Faisal University, Al-Ahsa 31982, Saudi Arabia; 2 Department of Botany and Microbiology, Faculty of Science, Cairo University, Cairo 12613, Egypt

**Keywords:** silver nanoparticles, *Trichophyton rubrum*, *Achillea santolina*, dermatophytosis

## Abstract

*Trichophyton rubrum* is the most common dermatophyte, and can cause cutaneous infections in humans and animals (dermatophytosis). In this study, we investigated the anti-dermatophytic potential of green synthesized silver nanoparticles using *Achillea santolina* extract (AS-AgNPs) in an in vitro and in vivo rat model of dermal *T. rubrum* dermatophytosis (TRD). The green synthesis of AS-AgNPs was performed using *A. santolina* extract and characterized by UV-VIS spectroscopy, zeta potential, imaging (transmission electron microscopy (TEM), X-ray diffraction (XRD), Fourier transform infrared spectroscopy (FTIR) and Energy dispersive X-ray analysis (EDX). The antifungal activity of AS-AgNPs was determined by the broth microdilution method, conidial germination, and hyphal growth inhibition. TEM and SEM were used to study the mode of the antifungal action of AS-AgNPs. AS-AgNPs inhibited the growth of *T. rubrum* with an MIC of 128 μg/mL, and suppressed the conidial germination and hyphal growth by 55.3% 84.6%, respectively. AS-AgNPs caused modified mycelial structures, increased cell membrane permeability, and cell wall damage. AS-AgNPs significantly increase the permeability of the fungal membrane, as revealed by reducing ergosterol biosynthesis. An increase in the intracellular ROS and the induction of apoptosis were also observed during AS-AgNP treatment. In addition, AS-AgNPs reduced the cell wall integrity, as shown by the reduction in the β-(1,3)-d-glucan synthase and chitin synthase activities. AS-AgNPs showed very low toxicity on primary human dermal fibroblasts (HDF) at the MIC. The topical treatment of the infected skin in the TRD rat model with AS-AgNPs showed a significant reduction in the fugal burden after 7 days and a complete clearance of fungal conidia, with a high recovery of epidermal and dermal structures after 14 days, compared to control rats. Interestingly, AS-AgNPs significantly attenuated the infiltrated inflammatory cells, in association with reducing the tissue proinflammatory cytokines including TNF-α, IL-1, IL-6, MOP and IL-17. In conclusion, our data prove AS-AgNPs to be a novel green topical therapy for dermatophytosis caused by *T. rubrum*.

## 1. Introduction

*Trichophyton rubrum* is the most prevalent filamentous fungus and causes most cases of cutaneous mycosis and onychomycosis [1,2]. Dermatophytic infections by *T. rubrum* involve the adhesion of arthroconidia to the stratum corneum, followed by keratin destruction [3,4]. Infection by dermatophytes induces the production of inflammatory cytokines in the skin and in the peripheral blood mononuclear cells of the host [5].

Topical and systemic synthetic antifungal drugs, including azoles, allylamines, itraconazole and terbinafine, are commonly used for the treatment of dermatophytosis. However, these medications need long-term adherence, and can cause drug resistance and toxicity [6,7]. Thus, there is a crucial need for developing novel anti-dermatophytic therapies with lower side effects.

The *Achillea* L. (Asteraceae) genus is present mostly in Asia and Europe and there are around 100 species in this genus [8]. *A. santolina*, (Qaysoom) grows as a herbal plant in the Mediterranean region and has traditionally been used as an antihemorrhagic and healing agent [9]. *A. santolina* was reported to be rich in flavones and polyphenols, and thus exerts antimicrobial and anti-inflammatory activities [10]. *A. santolina* is used in folk medicine to treat gastrointestinal disorders due its depurative, antispasmodic, and carminative properties [11]. In addition, *A. santolina* is used to treat hypoglycemia and is accepted as an alternative therapy for diabetes; this is due to its proven hypoglycemic activity in vitro and in vivo, due to the presence of several phenolic compounds [9,12]. However, no study has investigated the antifungal effect of *A. santolina* extracts against dermatophyte *T. rubrum*.

Nanoparticles are particles smaller than 100 nm in diameter. Nanomaterials can easily cross cellular barriers due to their special properties [13]. In nanotechnology, the biosynthesis of AgNPs attracted great attention due to their unique biophysical characteristics and improved biocompatibility, in addition to their significant biological properties in biomedical and environmental fields [14]. The physical and chemical-based synthesis of nanoparticles require the use of chemical-reducing agents, high temperature, and vacuum conditions, which are very expensive and hazardous to the environment [15]. Today, there is a crucial need to develop eco-friendly processes to synthesize nanoparticles with lower toxicity. Therefore, to avoid the disadvantages of physical and chemical synthesis methods, the plant extracts have been widely used in the green biosynthesis of AgNPs as reducing and stabilizing agents. Plants have promising significance, owing to the presence of diverse bioactive compounds, including flavonoids, phenolic acids, terpenoids, and alkaloids, which are useful for the biosynthesis of AgNPs to reduce silver ions for synthesizing the biomolecule of AgNPs [16].

We have recently shown the effective therapeutic potential of green biosynthesized nanoparticles, using plant extracts, in treating many fungal diseases. These include the effective inhibition of oral candidiasis by green AgNPs biosynthesized with *Erodium glaucophyllum* extract, *Lotus lalambensis* aqueous leaf extract, or the leaf extract of *Calotropis gigantean* [17,18,19], using *Artemisia sieberi* leaf extract against invasive pulmonary Aspergillosis [20].

In this report, we biosynthesized AgNPs, using *A. santolina* extract for the first time, and studied their anti-dermatophytic effect against *T. rubrum* in vitro and in vivo. Our data demonstrated the powerful antifungal activity of AS-AgNPs against *T. rubrum* in vitro by inhibiting its mycelial growth and affecting both cell wall and plasma membrane integrity. The topical treatment of dermatophytes in rats using AS-AgNPs was shown to significantly enhance skin healing by reducing the fungal burden and tissue inflammation.

## 2. Results

### 2.1. Biosynthesis and Characterization of AS-AgNPs

**Figure 1:**
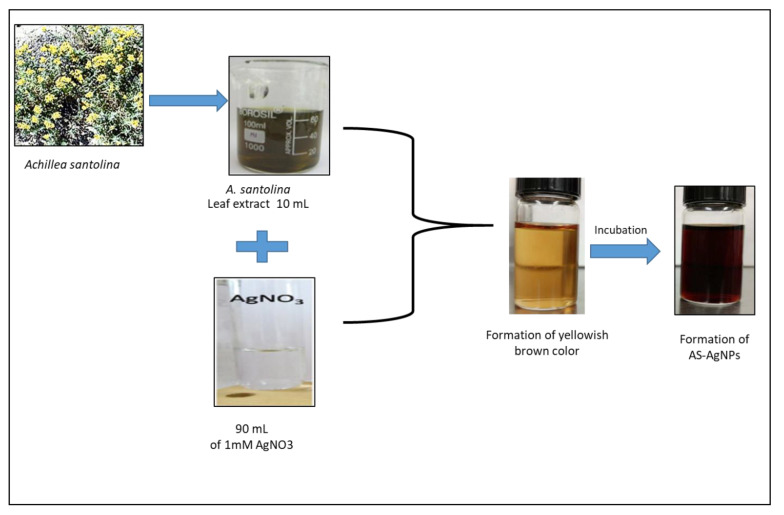
Biosynthesis of AS-AgNPs. Changes in color from yellowish brown to dark brown after the incubation of the *A. santolina* leaf extract and silver nitrate at room temperature in the dark.

**Figure 2:**
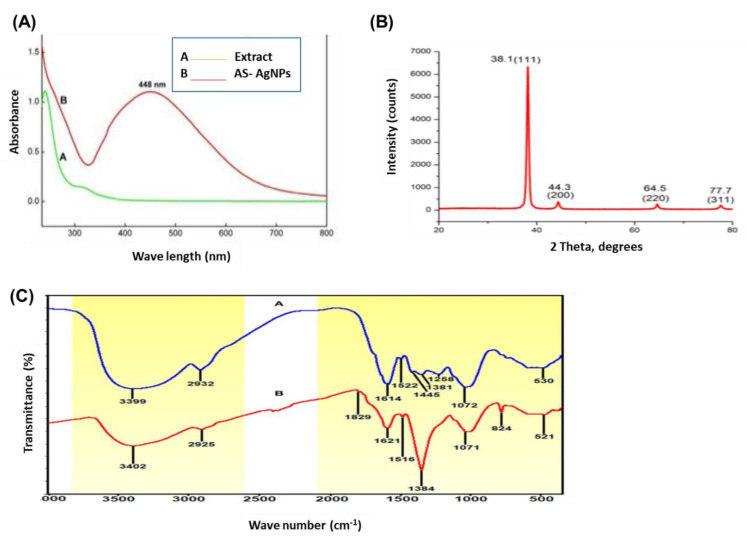
Verification of green biosynthesized AS-AgNPs. (**A**) UV–Vis spectrum of AS-AgNPs synthesized by *A. santolina* and *A. santolina* extract. The absorbance peak is at 448 nm, corresponding to the surface Plasmon resonance of AS-AgNPs (red line). Plant extract does not display any peak between 400 and 800 nm (green line). (**B**) The XRD spectrum of biosynthesized AS-AgNPs showed distinct diffraction peaks at 38.1°, 44.3°, 64.5°, 77.7°, and indexed at 2θ (degree) of (111), (200), (220), and (311) in face center cubic lattices. (**C**) FTIR spectrum of *A. santolina* extract: blue line, and AS-AgNPs synthesized by *A. santolina*: red line.

**Figure 3:**
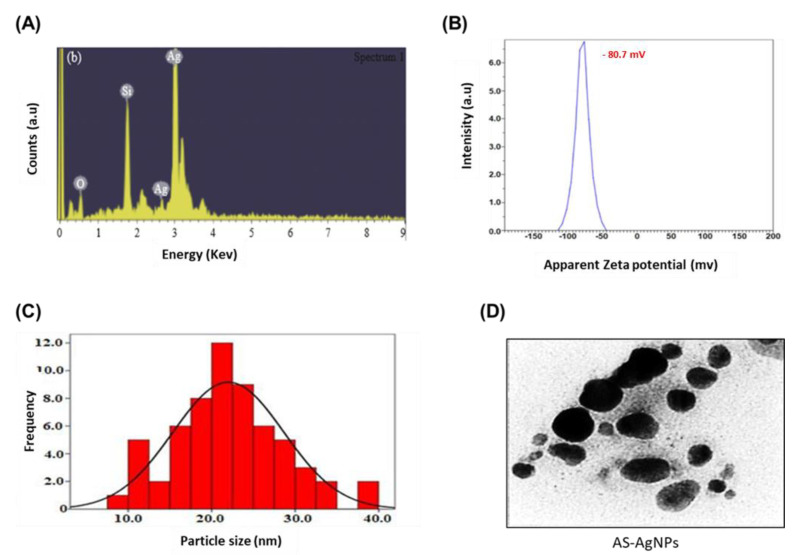
Characterization of green biosynthesized AS-AgNPs. (**A**) The EDX spectrum of silver nanoparticles synthesized by *A. santolina* showed a pattern of approximately 63.65% occurrence of Ag metal. (**B**) Zeta potential of silver nanoparticles synthesized by *A. santolina*. The value of zeta potential is −80.7 mV. This value displays the high stability of *A. santolina* extract to synthesize AS-AgNPs. (**C**) The size distribution histogram of the dynamic light scattering (DLS) analysis of the biosynthesized AS-AgNPs. According to DLS analysis, this size distribution of AS-AgNPs is centered at 21.9 nm. (**D**) TEM micrograph of AS-AgNPs biosynthesized from *A. santolina* leaf extract. AS-AgNPs appeared spherical in shape with an average size of 21.9 nm.

The change in color of AS-AgNPs, from colorless to dark brown, demonstrates the synthesis of silver nanoparticles (Figure 1). This change is attributed to the excitation of surface Plasmon resonance (SPR) within the biosynthesis of AgNPs. The UV–Vis absorption spectrum of AS-AgNPs showed a strong peak at 448 nm, which is due to the SPR of AgNPs (Figure 2A, red line). A small peak also appeared at approximately 350 nm. The appearance of two peaks shows that the particles are of different sizes. The peak at 350 nm is for smaller nanoparticles. On the other hand, the UV–Vis spectra does not display any evidence of absorption in the range of 400–800 nm for the plant extract (Figure 2A, green line). The crystalline nature of AS-AgNPs is examined by the XRD technique. AS-AgNPs showed four different peaks at 2θ = 38.1, 44.3, 64.5, and 77.7. All peaks corresponded to standard Bragg reflections (111), (200), (220), and (311) of the face center cubic lattice (Figure 2B). The FTIR spectrum for *A. santolina* extract showed absorption bands at 3399, 2932, 1614, 1522, 1445, 1381, 1258, 1072, and 530 cm^−1^ (Figure 2C, blue line). The peaks ranging from 3200 to 3600 cm^−1^ are attributed to the hydroxyl and amine stretching vibrations in the *A. santolina* extract. The peak at 2932 cm^−1^ is related to C-H stretching. The peak at 1614 cm^−1^ is assigned to NH bending. The peak at 1522 cm^−1^ is related to the aromatic ring of the terpenoids. The carboxyle group is verified by the peak at 1445 cm^−1^. The peak at 1381 cm^−1^ is assigned to the CH bending of aldehyde groups. The peak at 1258 cm^−1^ is related to the stretching of ester [21]. The peak at 1072 cm^−1^ is associated with C-O stretching. The peak at 530 cm^−1^ corresponds to the bonding of oxygen with the hydroxyl groups. However, the FTIR spectrum for the biosynthesized AS-AgNPs revealed that the peaks at 3399, 2932, 1522, and 530 cm^−1^ shifted to 3402, 2925, 1516, and is assigned to hydroxyl and amine stretching, C-H, C=O stretching, the aromatic ring of the terpenoid, and AgO, respectively [22,23]. The appearance of a new peak at 1829 cm^−1^ corresponds to a carboxylate group. The new peak at 823 cm^−1^ increased, suggesting that C-H groups could also bonded with the AS-AgNPs. The disappearance of some peaks at 1445 and 1258 cm^−1^ has been shown in the AS-AgNPs FTIR spectrum. The peak intensity at 1621 cm^−1^ decreased, signifying the association of N-H bending. The peak intensity at 1384 cm^−1^ increased, suggesting the C-H bending of aldehyde groups (Figure 2C, red line). The EDX pattern designates a 63.8% occurrence of silver metal. Additionally, the EDX spectrum revealed the presence of an oxygen peak that could be coming from the chamber of EDX (Figure 3A). AS-AgNPs were found to carry a negative zeta potential of −80.7 mV, which defines the repulsion between AgNPs and increases the stability of the preparation (Figure 3B). The hydrodynamic diameter of the AS-AgNPs is determined by the DLS technique (Figure 3C). Regarding the size and morphology of the AgNPs, TEM and DLS analyses were performed. According to DLS analysis, the size distribution of AS-AgNPs is centered at 21.9 nm. AS-AgNPs were mostly spherical in shape, with an average size of 21.9 nm, as assessed by TEM (Figure 3D). Therefore, TEM and DLS results, as a rule, are in agreement with each other.

### 2.2. In Vitro Antifungal Activity of AS-AgNPs against T. rubrum

**Figure 4:**
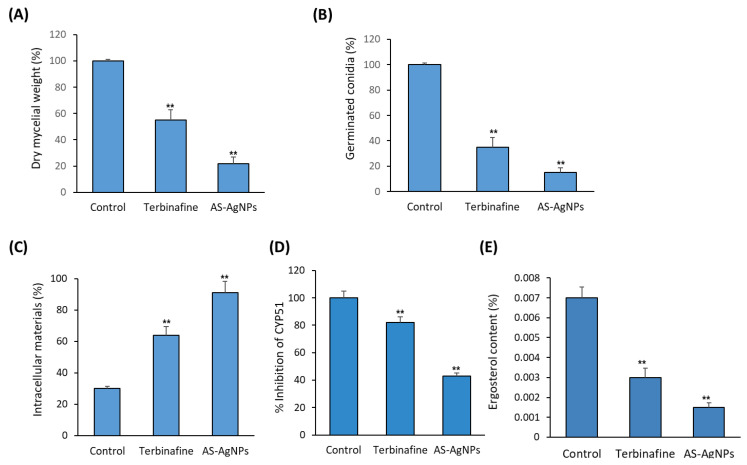
Effect of AS-AgNPs on mycelial growth and cell membrane integrity of *T. rubrum*. (**A**) Percentage of dry mycelial weight produced by *T. rubrum* in the absence (control) and presence of terbinafine (MIC: 256 μg/mL) and AS-AgNPs (MIC: 128 μg/mL). (**B**) Percentage of conidial germination of *T. rubrum* in the absence (control) and presence of terbinafine (256 μg/mL) and AS-AgNPs (128 μg/mL). (**C**) Rate of release of intracellular material from *T. rubrum* at 260 nm in the absence (control) and presence of terbinafinel (MIC: 256 μg/mL) and AS-AgNPs (MIC: 128 μg/mL). (**D**) Inhibition rate (%) of CYP51 enzyme in *T. rubrum* in the absence (control) and presence of terbinafinel (MIC: 256 μg/mL) and AS-AgNPs (MIC: 128 μg/mL). (**E**) Ergosterol content as percent of the wet weight of *T. rubrum* in the absence (control) and presence of terbinafinel (MIC: 256 μg/mL) and AS-AgNPs (MIC: 128 μg/mL). Values are mean ± SD of three independent experiments (** *p* < 0.05 compared to control non-treated cells).

The results of the broth microdilution assay revealed that the MIC of AS-AgNPs against *T. rubrum* was 128 μg/mL; meanwhile, the MIC value of terbinafine (positive control) was 256 μg/mL. Additionally, we compared the antifungal activity of plant extract and AS-AgNPs using a disk diffusion method. The AS-AgNPs displayed a higher antifungal action where the diameter of the inhibition zone (IZD) was 38 mm, although the extract showed a lower antifungal activity with an IZD of 25 mm (Supplementary Table S1). The MIC values of AS-AgNPs and plant extract were 128 and 512 µg/mL, respectively. As shown in Figure 4A,B, AS-AgNPs displayed a higher inhibitory effect on the mycelial growth of *T. rubrum* than terbinafine, as assessed by measuring dry mycelial weight and conidia germination (Figure 4A,B).

### 2.3. In Vitro Studies on the Antifungal Mechanism of AS-AgNPs

**Figure 5:**
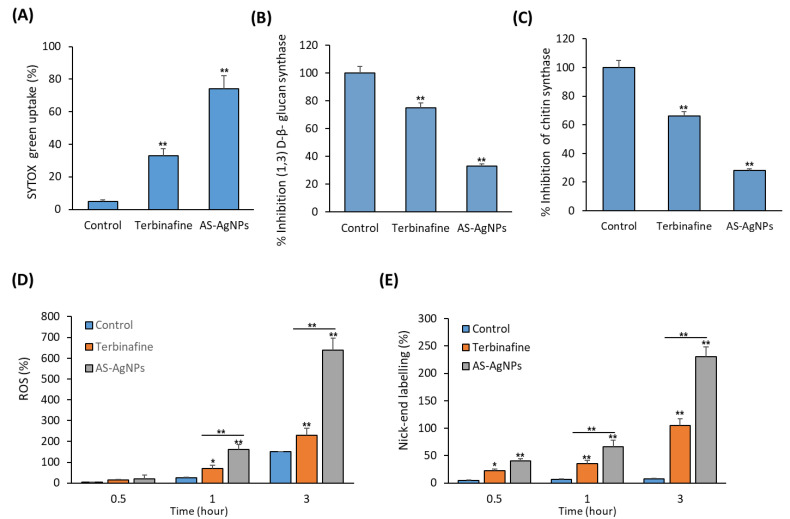
In vitro mechanistic studies of antifungal action of AS-AgNPs against *T. rubrum*. (**A**) Effect of AS-AgNPs (128 μg/mL) on SYTOX^®^ Green uptake into *T. rubrum* after 24 h of treatment. (**B**) % Inhibition of β-(1,3)-d-glucan synthase in the absence or presence of AS-AgNPs (128 μg/mL) against. *T. rubrum*. (**C**) % Inhibition of chitin synthase in the absence or presence of AS-AgNPs (128 μg/mL) against *T. rubrum*. (**D**) Effect of AS-AgNPs (128 μg/mL) on ROS release from *T. rubrum* after treatments of different durations. (**E**) Effect of AS-AgNPs (128 μg/mL) on the nick-end labeling of *T. rubrum* after treatments of different durations. Values are mean ± SD of three independent experiments (* *p* < 0.05, ** *p* < 0.05 compared to control non-treated cells).

To study the mechanism underlying the antifungal activity of AS-AgNPs against *T. rubrum*, the effect of As-AgNPs and terbinafine on the cell membrane integrity of *T. rubrum* were compared. As shown in Figure 4C, AS-AgNPs significantly increased the leakage of intracellular material by 200% and 33%, when compared to the control and terbinafine, respectively, after an 8 h incubation. In addition, AS-AgNPs significantly inhibited CYP51 (Cytochrome P450 Family 51), a mediator of the synthesis of ergosterol (A sterol that acts to maintain cell membrane integrity) as well as ergosterol production by 51% and 47%, respectively, when compared with terbinafine at the MIC (Figure 4D,E). In this context, AS-AgNPs at the MIC increased the uptake of SYTOX^®^ Green, which determines the cell viability by 74.3% in *T. rubrum*; meanwhile, terbinafine increased this uptake by only 37.5%, when compared to the control (Figure 5A). Thus, AS-AgNPs might inhibit the growth of *T. rubrum* by changing its membrane permeability.

The measurement of β-(1,3)-d-glucan synthase and chitin synthase activities were used to assess the effect of AS-AgNPs on the integrity of the fungal cell wall in *T. rubrum*. Interestingly, AS-AgNPs significantly inhibited the activity of β-(1,3)-d-glucan synthase and chitin synthase by 71% and 76%, respectively, when compared to the control (Figure 5B,C). As shown in Figure 5D, AS-AgNPs showed a time-dependent stimulatory effect on ROS production by *T. rubrum* compared to terbinafine, when used at the MIC (Figure 5D). A TUNEL assay for DNA fragmentation showed the time dependent stimulatory effect of AS-AgNPs on the level of nick-end labeling, when compared to the control (Figure 5E).

### 2.4. Effect of AS-AgNPs on Ultrastructural Changes in T. rubrum

**Figure 6:**
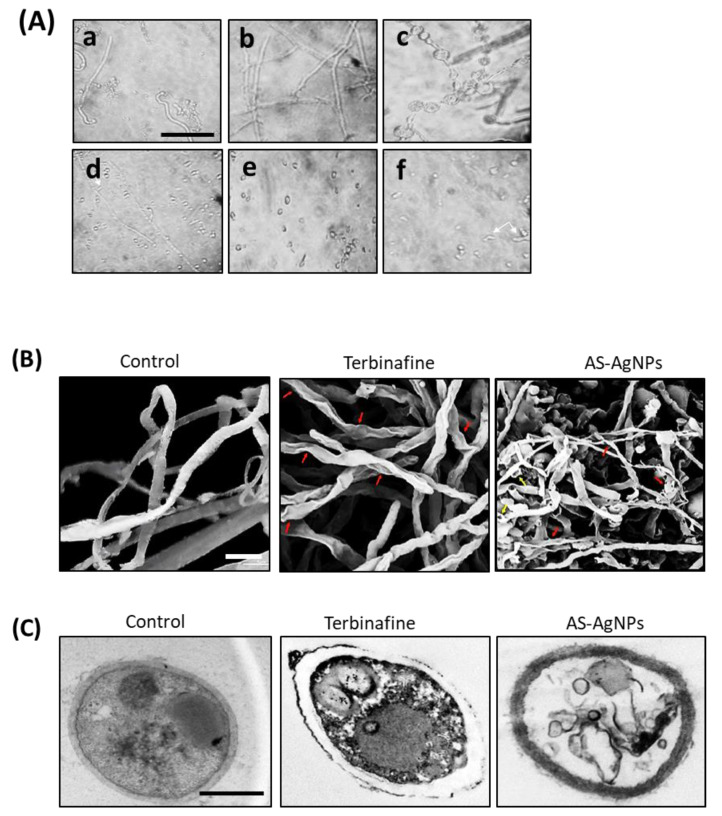
Effect of AS-AgNPs on cell morphology of *T. rubrum*. (**A**) Inverted phase-contrast microscope images of *T. rubrum* with different treatments. (**a**) Saline-treated fungus display germination that started 4 h subsequent to the incubation of spores. (**b**) Normal structure of hyphae after 72 h incubation. (**c**) Terbinafine-treated cells (256 μg/mL) displaying different modifications; thick broader hyphae with vacuoles, tiny elongated hyphae with tear-shaped microconidia (arrow) (**d**). (**e**) AS-AgNP-treated cells (128 μg/mL) showed inhibition of spore germination. There are malformations, cellular injuries, and dumpy necrotic hyphae (arrow) (**f**). 400× (bar 100 µm). (**B**) SEM of *T. rubrum* treated with saline, terbinafine (256 μg/mL), and As-AgNPs (128 μg/mL). The saline-treated sample (negative control) displays normal tubular and uniform hyphae. The terbinafine–treated cells (positive control) show minor distortion in the shape of the cells and collapse in cell wall (red arrows); however, significant modifications are obvious in the case of AS-AgNP-treated cells where there are the fragmentation of cells into flat shapes (red arrows) and the leakage of intracellular material after damage (yellow arrows). Bar = 2.5 μm. (**C**) TEM of *T. rubrum* treated with saline, terbinafine, and As-AgNPs. The fungal hyphae displayed morphological alterations once subjected to terbinafine and As-AgNP. *T. rubrum* treated with terbinafine showed damage of cytoplasm content and the cell membrane started to separate itself from the cell wall. Meanwhile, the treatment of *T. rubrum* with AS-AgNPs resulted in the complete destruction of the fungal hypha through lysis, and the cell wall and compartment were completely damaged. Bar = 250 nm.

**Figure 7:**
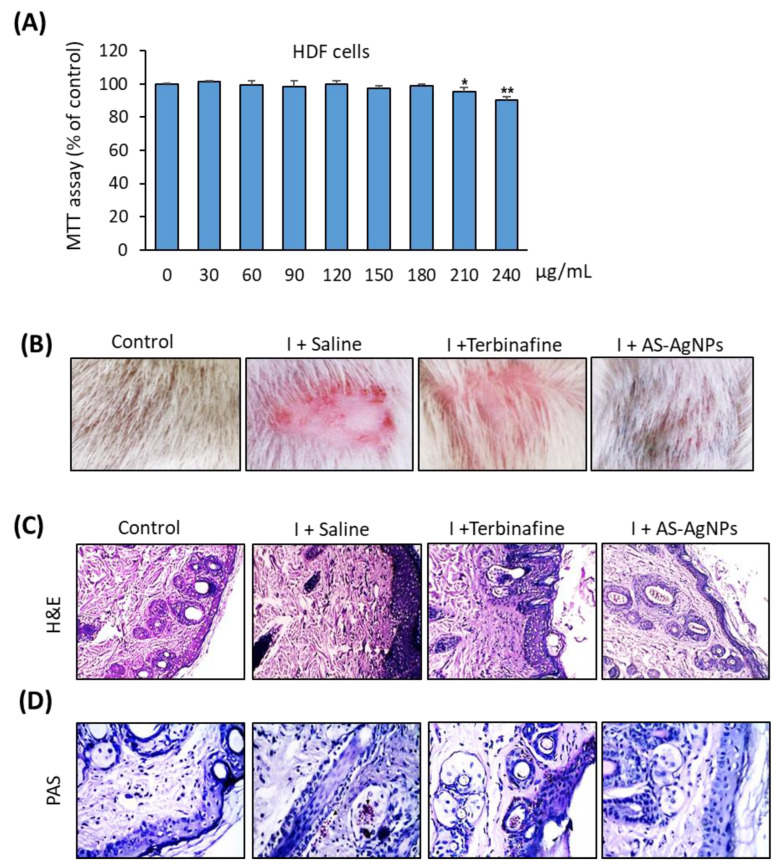
Skin histological analysis of rat dermatophytosis model. (**A**) Cytotoxicity of AS-AgNPs on primary HDF cells. Cell viability was measured by MTT assay after 48 h of incubation at different concentrations of AS-AgNPs. Values are mean ± SD of three independent experiments (* *p* < 0.05, ** *p* < 0.05 compared to control non-treated cells). (**B**) Representative images of infected skin tissue taken on day 14 post treatment. Groups are as follows: control (non-infected), I + Control (infected rat with saline treatment), I + Terbinafine (infected rat treated with terbinafine (10 mg/kg) and I + AS-AgNPs (infected rat treated with AS-AgNPs (0.5 μg/g)). Skin tissue sections stained with H&E (**C**) and periodic acid–Schiff (PAS) (**D**). I + Terbinafine displayed slight effect on thickness of epidermis of skin, and fungal fragments were still identified in the epidermis and the hair follicle (PAS). The AS-AgNP-treated group showed complete recovery of skin structure (H&E) with an absence of fungal cells.

An inverted microscopic investigation showed normal cell structures with consistent cytoplasm, and no spores or chlamydospores; in addition long septate hypha were revealed in untreated mycelium of *T. rubrum* (Figure 6(Aa,b)). Treatment of *T. rubrum* with terbinafine (256 µg/mL) led to the formation of smaller and broader hypha with several vacuole-like structures inside them and the production of thinner branched hypha with tear-shaped microconidia (Figure 7(Ac,d)). Interestingly, AS-AgNPs (128 µg/L) inhibited the germination of spores completely, and resulted in many malformations of and severe injury to the fungal structures of *T. rubrum* (Figure 6(Ae,f)).

SEM images of the *T. rubrum* treated with AS-AgNPs showed more visible malformed cells, the folding of fungal hypha, an increased cell membrane permeability, and the formation of round swells; these are amongst the greatest significant detected modifications. On the other hand, the *T. rubrum* treated with terbinafine demonstrated cell wall breakdown, which seemed to include scattered hypha and filamentous hyphae distorted into flat shapes (Figure 6B).

Figure 6C displays the TEM images of the *T. rubrum* hyphae cultured on Sabaroud dextrose agar medium. The *T. rubrum* hyphae treated with terbinafine showed severe cytoplasm damage and a condensed cell wall. In addition, the cell membranes started to break from each other. AS-AgNP treatment was shown to destroy hyphae by lysis and to destroy the cell wall completely. In addition, the cytoplasmic compartment also displayed desolation and fragmentation. In general, AS-AgNPs result in severe injury to the cell wall and organelles.

### 2.5. Effective In Vivo Topical Treatment of Dermal T. rubrum Infection Using AS-AgNPs

**Figure 8:**
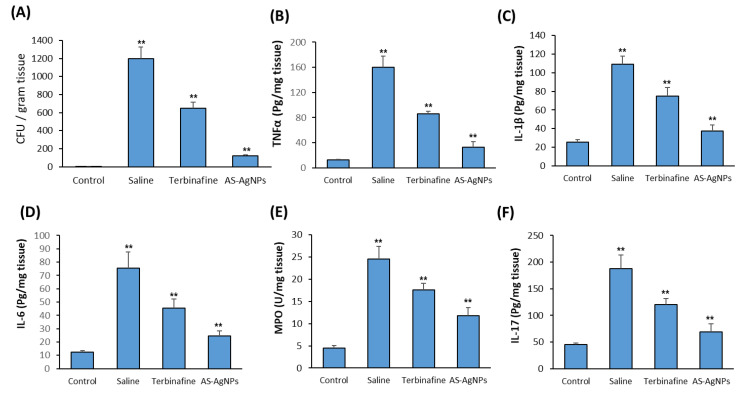
Effect of AS-NPs on fungal burden and dermal pro-inflammatory cytokines in dermatophytosis rat model. (**A**) Inhibitory effect of AS-AgNPs versus trebinafine treatment on colonization of *T. rubrum* in infected skin homogenate after 14 days of treatment. Infected rats with *T. rubrum* were treated for 14 days with saline, terbinafine (10 mg/kg) or topically with AS-AgNPs (0.5 μg/g. Effect of AS-AgNPs versus trebinafine treatment on the pro-inflammatory cytokines produced in the infected skin tissues of the dermatophytosis rat model. The following cytokines, including (**B**) TNF-α, (**C**) IL-1β, (**D**) IL-6, (**E**) MOP and (**F**) IL-17, were measured as described in M&M. Values are expressed as means ± SD (n = 6 rats/group) (** *p* < 0.005, compared to control non-infected cells).

Before applying AS-AgNPs in vivo, we examined their cytotoxicity on primary human dermal fibroblasts (HDF). As shown in Figure 8A, AS-AgNPs displayed no cytotoxicity on HDF cells at the MIC of 128 µg/mL and up to the concentration of 150 μg/mL. AS-AgNPs started to show significant cytotoxicity at 200 μg/mL.

A photographic and histopathological assessment of H&E-stained skin samples showed a normal skin structure, a suitable number of normal hair follicles, and sebaceous glands in the control non-infected rat (Figure 7B,C). In contrast, the infected untreated skin samples showed an increased epidermal thickness and hyperkeratosis, with a reduced number of hair follicles. In addition, the infiltration of inflammatory cells into the dermal layer was also observed (Figure 7C). Interestingly, the topical treatment of infected skin with AS-AgNPs displayed a significant improvement in skin structure repair over using the terbinafine treatment (Figure 7A). The AS-AgNP-treated group showed no significant proliferation of the epidermis and stratum corneum hyperkeratosis, while a mild thickening and proliferation of the epidermis with a low inflammatory response were detected in the terbinafine-treated group. In addition, PAS staining confirmed the absence of fungal infection in the AS-AgNP-treated group, when compared to the infected non-treated group (Figure 7D). On the other hand, the terbinafine treatment group still displayed a PAS-positive reaction for fungal burden (Figure 7D).

### 2.6. AS-AgNPs Significantly Reduce Fungal Burden and Tissue Inflammation in Rat Model of T. rubrum Deramatophytosis

To study the effect of AS-AgNPs on the fungal burden in vivo, we quantify the colonization of *T. rubrum*, which is isolated from infected skin with and without treatment. As shown in Figure 8A, the treatments with AS-AgNPs and terbinafine significantly inhibit the mean fungal burden by 84% and 43%, respectively, in comparison to the saline-treated group (Figure 8A). Thus, the topical treatment with AS-AgNPs displayed a higher antifungal activity than terbinafine.

We also studied the effect of AS-AgNPs on deramatophytosis-induced inflammation by measuring pro-inflammatory cytokines in dermal tissues from different groups. Consistent with its inhibitory effect on fungal burden, the AS-AgNP treatment significantly reduced the elevated levels of pro-inflammatory cytokines, TNF-α, IL-1, IL-6, MOP and IL-17 by 76%, 64%, 68%, 52%, and 65%, respectively, in comparison to the saline-treated group (Figure 8B–F). In addition, the anti-inflammatory effect of AS-AgNPs was more pronounced than the effect of terbinafine on all the tested cytokines by approximately 20–14% (Figure 8B–F).

## 3. Materials and Methods

### 3.1. Plant Material and Preparation of the Extract

*A. santolina* were collected during the month of May from Saudi Arabia, Al-Hassa-Damam road, Eastern Province. In total, 10 gm of *A. santolina* leaves were dispensed in 100 mL of sterilized distilled water and boiled for 60 min at 95 °C. The extract was filtered, evaporated by a rotary vacuum evaporator at 40 °C, and stored at 4 °C [24].

### 3.2. Biosynthesis of AS-AgNPs

AgNO_3_ solution was prepared in deionized water and used for the biosynthesis of AS-AgNPs. In total, 10 mL of *A. santolina* leaf extract was added to 90 mL of 1 mM of AgNO_3_ and kept in a water bath at 90 °C for 60 min for the reduction process of silver ions [25]. The color change from colorless to dark brown denotes the biosynthesis of silver nanoparticles.

### 3.3. Characterization of AS-AgNPs

A UV–Vis spectrophotometer(Perkin Elmer, Boston, MA, USA) from 200 to 900 nm functioned at a resolution of 1 nm, and was used as a function of wavelength for the spectral analysis of AS-AgNPs. The crystalline nature of AS-AgNPs was determined by a XDL 3000 powder X-ray diffractometer (XRD, Unisantis XMD-300, Malvern Panalytical, Malvern, UK ). The surface morphology and size of AS-AgNPs were observed by transmission electron microscopy (TEM) (JEM-2010; JEOL, H7100; Hitachi Ltd., Tokyo, Japan) at an accelerating voltage of 200 kV. The spectra of the AgNPs were measured by Fourier transform infrared spectroscopy (FTIR) (PerkinElmer, Waltham, MA, USA) at a resolution of 4 cm^−1^ with KBr pellets. AS-AgNPs were also subjected to energy dispersive X-ray analysis (EDX), according to the method of [26]. The size distribution of the particles was determined by measuring the dynamic oscillations of light scattering intensity (DLS), resulting from the Brownian motion of the particles using the Zetasizer Nano ZS, Malvern Instruments Ltd., Malvern, UK. Zeta potential was used to study the stability of the AgNPs. The measurements were performed in the first 3–4 h after the synthesis of AgNPs and then once a week for 4 weeks.

### 3.4. Fungal Strain

*T. rubrum* ATCT 9322 (ATCT, Kasr Al-Ainy, Cairo, Egypt) was used throughout this study. *T. rubrum* was cultured at 28 °C on modified Sabouraud dextrose agar (MSDA) slants containing the following: peptone 10 g/L, glucose 40 g/L and agar 15 g/L at a pH range of 5.4–5.8.

### 3.5. Minimum Inhibitory Concentration (MIC)

The broth microdilution assay was used to measure the MIC of the AS-AgNPs and terbinafine. MSDA was added to all the wells of 96-well plates. Serial bifold dilutions of the two antifungal agents were prepared to obtain concentrations between 1 μg/mL and 512 μg/mL. Lastly, 10 μL aliquots of the *T. rubrum* suspension (1 × 10^6^ CFU/mL) were added to the wells and the plates were incubated at 28 °C for 7 days. The MIC was described as the lowest concentration able to visually inhibit 100% of the fungal growth [27].

### 3.6. Hyphal Growth Inhibition

The effect of AS-AgNPs and terbinafine on mycelial growth were assessed by measuring the dry mycelial weight of *T. rubrum* according to the method [28]. Briefly, MSDA, (5 mL) with the antifungal drugs (at their MIC values), was added to 0.5 mL of the fungal suspension (1 × 10^6^ CFU/mL). The samples were incubated at 28 °C for 10 days and the fungal mycelia were filtered using sterile filter paper and dried at 65 °C for 15 min. The dry fungal mycelia were then weighed and the percentage of mycelial production was measured, using the growth in the control test tubes as 100% of potential dry mycelia weight.

### 3.7. Effects on Conidial Germination

MSDA (500 μL), containing the antifungal drugs at their MIC values, was mixed with 500 μL of fungal suspension (1 × 10^6^ CFU/mL) and then incubated at 28 °C. After 24 h, the number of germinated and ungerminated conidia were measured using a hemocytometer where the percentage of germinated conidia was determined.

### 3.8. Membrane Permeability Assays

#### 3.8.1. Release of Intracellular Material

In total, 2 mL aliquots of the fungal suspension (1 × 10^6^ CFU/mL) were added to 9 mL of MSDA containing either AS-AgNPs or terbinafine, and incubated for 8 h. Then, fungal cells were centrifuged at 3,000 rpm for 5 min, and the absorbance of the supernatant was measured at 260 nm with a UV-visible Spectrophotometer (Shimadzu, Kyoto, Japan.). An alcoholic potassium hydroxide solution was used as a reference compound as it results in 100% cellular leakage. The rate of release of intracellular material absorbing at 260 nm was determined by comparing the test values with the lysing agent (100%) [29].

#### 3.8.2. Ergosterol Quantitation

In total, 1 mL of *T. rubrum* inoculum (1 × 10^6^ CFU/mL) was added to 9 mL of MSDA containing either AS-AgNPs or terbinafine and incubated for 5 days at 28 °C. Then, the fungal cells were centrifuged at 3,000 rpm for 5 min, washed with sterile distilled water and the wet weight of the fungal pellet was measured. In total, 3 mL of lysing agent was added to each pellet and vortexed for 1 min. Fungal suspensions were incubated at 90 °C for 1 h and allowed to cool. Ergosterol was extracted by adding a mixture of 3 mL of n-heptane, then vortexing for 5 min. The heptane layer was transferred to Eppendorf tubes and stored in a refrigerator for one day. Aliquots of ergosterol extracts were determined by measuring the absorbance at 281.5 nm with a UV-Visible spectrophotometer (Shimadzu). Ergosterol content was measured as a percentage of the wet weight of the fungal cell, as described by [30].

#### 3.8.3. Activity of CYP51 Enzyme

Fungal suspension (1 × 10^6^ CFU/mL) was prepared as previously described. The fungal cells, treated with either AS-AgNPs or terbinafine (MIC) for 5 days, were collected by centrifugation, and the enzyme was extracted by ultrasonication in an ice bath. The enzyme activity was determined by the ELISA quantitative detection kit (SinoBestBio, Shanghai, China), according to the method of [31].

#### 3.8.4. SYTOX^®^ Green Uptake

This experiment was performed by observing the uptake of SYTOX^®^ Green, which is a high-affinity nuclear stain able to enter the cells with compromised membranes [32]. *T. rubrum* cells were incubated with AS-AgNPs, terbinafine, or phosphate buffer saline (negative control) at 37 °C for 24 h. SYTOX^®^ Green (0.5 µM) was added to the fungal cultures for 10 min and the fluorescence produced by the fungal cells was detected using a fluorescence spectrometer (SpectraMax i3 Multi-Mode Detection Platform, Molecular Devices, LLC, Sunnyvale, CA, USA) at an excitation wavelength of 488 nm and an emission wavelength of 540 nm.

### 3.9. Cell Wall Integrity Assays

#### Activity of β-(1,3)-d-Glucan Synthase and Chitin Synthase

The fungal suspension (1 × 10^6^ CFU/mL) was treated with either AgNPs or terbinafine (MIC), as previously described. The samples were incubated at 28 °C, with shaking for 7 days. Then, the samples were collected, washed with PBS, centrifuged, and the deposits were collected. In total, 0.2 g of fungal deposit was added to 2 mL of extracting solution, sonicated on an ice bath, placed at −20 °C overnight, and repetitively frozen and thawed to extract the enzymes. The samples were then centrifuged at 5000× *g* for 5 min at 4 °C and the supernatants were collected. The activity of β-(1,3)-d-glucan synthase and chitin synthase were measured using a quantitative detection kit (SinoBestBio, Shanghai, China). The enzyme activity was determined by measuring the absorbance at 550 nm (β-(1,3)-d-glucan synthase) and 585 nm (chitin synthase) with a microplate reader, following the method of [31].

### 3.10. Reactive Oxygen Species (ROS) Assay

The fluorogenic probe 5-(and-6)carboxy-2′,7′-dihydrodichlorofluorescein diacetate (carboxy-H2DCFDA) was applied to detect ROS production in the *T. rubrum* cells following the method described previously [33]. *T. rubrum* cells were incubated for 3 h with AS-AgNPs or terbinafine. The fluorescence released by the fungal cells was measured by a fluorescence spectrometer at an excitation wavelength of 488 nm and an emission wavelength of 540 nm.

### 3.11. DNA Fragmentation Assay

A TUNEL assay was performed to identify the presence of any DNA fragmentation according to the method described previously [34]. *T. rubrum* cells were incubated with AS-AgNPs or terbinafine at their MIC values for 3 h. Cells were washed with PBS and fixed with 4% paraformaldehyde in PBS for 1 h at 20 °C. Next, the cells were washed with PBS and incubated in ice for 2 min with the permeabilization solution as previously described. Subsequently, the cells were washed with PBS and labeled using an In Situ Cell Death Detection Kit (Thermo Fisher Scientific, Bremen, Germany), according to the manufacturer’s instructions. Finally, 50 µL of the TUNEL reaction mixture was added to the fungal cells, and the resultant mixture was incubated at 37 °C for 60 min in the dark. The fungal cells were then washed with PBS and observed by a fluorescence spectrometer.

### 3.12. Effects on Morphology and Ultrastructure

#### 3.12.1. Inverted Phase Contrast Microscopy

The morphological alterations to *T. rubrum* after treatment with the antifungal drugs were determined in a 96-well microtiter plate with flat bottom. About 1 × 10^6^ CFU/mL of the *T. rubrum* was prepared and treated with AgNPs or terbinafine at their MIC values in RPMI medium without phenol red, and incubated at 28 °C for 3 days before their observation under a phase-contrast inverted microscope (×400). DMSO-treated *T. rubrum* was used as a negative control. Changes identified in the fungal structures were compared with the normal growth in the negative control [35].

#### 3.12.2. Scanning and Transmission Electron Microscopy

The morphological modifications of fungal structures were also evaluated in AgNP-treated and untreated samples using scanning (SEM, Hitachi High-Technologies Europe GmbH, Krefeld, Germany) and transmission (TEM, JEOL-JEM 1400, Freising, Germany) electron microscopy [36]. Briefly, *T. rubrum* cells were incubated with AgNPs or terbinafine at their MIC values as described previously. The fungal mat was centrifuged and the cell pellet was kept in glutaraldehyde solution (Agar Scientific, Essex, UK) for 24 h then rinsed with PBS. The cells were then fixed and stained by osmium tetroxide (Agar Scientific, Essex, UK), dehydrated by graded ethanol series and finally submerged in ethanol for one hour. For SEM examination, the final drying was carried out at room temperature by hexamethyldisilazane (Merck, Darmstadt, Germany) for 25 min. Sputter coating of the samples was performed with gold. SEM images were obtained by Field-Emission SEM, Hitachi S-5500 (Hitachi High-Technologies Europe GmbH, Krefeld, Germany). For TEM examination, samples were post-fixed by osmium tetroxide and implanted in epoxy resin (Agar Scientific, Essex, UK) overnight. Samples were sectioned by Ultra-microtome (Leica Camera AG, Wetzlar, Germany)) and stained with 2% uranyl acetate (Agar Scientific, Essex, UK) and 4% lead citrate (Agar Scientific, Essex, UK). The transmission electron micrographs were taken by EM208S (Philips, TSS microscopy, Hillsboro, USA) 100 kV).

### 3.13. Cell culture and Cytotoxicity Assay

The human primary dermal fibroblast HDFa cell line was obtained from (ThermoFisher Scientific, Waltham, MA, USA) (C0135C). Cells were cultured in Dulbecco’s modified Eagle’s medium (DMEM; Sigma-Aldrich, St. Louis, MO, USA), supplemented with 1% penicillin/streptomycin (Gibco Invitrogen, Carlsbad, CA, USA) and 10% heat-inactivated fetal bovine serum (FBS) (Sigma-Aldrich).

Cell viability was measured by using a MTT cell proliferation assay kit (Sigma-Aldrich), according to the manufacturer’s instructions. HDF cells were cultured in 96-well plates and treated with AS-AgNPs (0–200 µg/mL) for 48 h. The cultured medium was replaced by medium containing 0.5 mg/mL MTT to metabolize to formazan. An ELISA plate reader was used to measure the optical density at 550 nm, as described [18].

### 3.14. Rat Dermatophytosis Model

The in vivo animal experiment was approved and licensed by the Research Ethics Committee, Deanship of scientific research at King Faisal University (KFU-REC-2022-OCT-ETHICS218). Male albino rats (n = 24, 3 months old) were housed under conventional conditions of 25 ± 2 °C and 12 h dark/light cycle, with a standard diet and water ad libitum.

Rats were exposed to common anesthesia intramuscularly by a 0.2 mL combination of ketamine, xylazine, and acepromazine (3:3:1, by volume). An area of 2 × 2 cm^2^ on the back of the mice was shaved and the skin inside this area was scraped using sterile fine grit sand paper. *T. rubrum* conidia (1 × 106 cells/animal) was applied in a 100 μL volume of medium/animal and smoothly scrubbed on the grazed skin. The control rates were inoculated with 100 μL of PBS. Rats were divided into 4 groups (n = 6 ) as follows: group 1, non-infected and non-treated (negative control group); group 2, infected animals treated with saline only; group 3, infected animals treated daily intramuscularly with terbinafine (10 mg/kg) and group 4, infected animals treated topically with AS-AgNPs (0.5 μg/g) every 5 days. The course of the treatment continued for 14 days.

### 3.15. Fungal Burgen

The determination of the fungal burden was performed on day 7 and day 14 post treatment by gathering a dermal biopsy from anesthetized animals using ketamine/xylazine (80/15 mg/kg). Skin biopsy was suspended in PBS and plated on PDA medium. The results were expressed as colony-forming units (CFU) per gram of skin.

### 3.16. Histopathology

Rats were sacrificed on day 14 post treatment, and the skin areas with infection were fixed in buffered 10% formalin (*v*/*v*) and embedded in paraffin. Skin samples were cut into 5-µm-thick sections and stained with (H&E) and Periodic acid–Schiff (PAS). Nikon 80i light microscope (Nikon Corporation, Tokyo, Japan) was used to take tissue section images.

### 3.17. Measurement of Cytokine Levels in Skin Tissue

TNF-α, MOP, IL-1, IL-6, and IL-17 were measured by the enzyme-linked immunosorbent assay (ELISA) kit (MyBioSource, Inc., San Diego, CA, USA) in skin tissues according to the manual instructions and as described previously [20,37]. In brief, 500 μg of skin tissue was homogenized in 1.0 mL of PBS, centrifuged at 3000× *g* for 10 min at 4 °C, and the supernatants were collected for measurements. Data were expressed as picograms per milligram of tissue.

### 3.18. Statistical Analysis

All values were expressed as the mean ± SD (standard deviation) of at least 3 independent experiments. Power calculation was performed for 2 samples using the unpaired Student’s T-test (2-tailed), assuming equal variation in the two groups. Differences were considered statistically significant at * *p* < 0.05, and ** *p* < 0.005.

## 4. Discussion

This report provides an alternative topical treatment for dermatophyte *T. rubrum*, using green biosynthesized AgNPs with *A. santolina* extract.

In this report, we biosynthesized AS-AgNPs for the first time using the water extract of *A. santolina* as a bioreductant, which increases its biocompatibility and pharmacological properties. Consistent with our technique, it has been previously described that, once the AgNPs are formed in a reaction mixture, the color changes to dark brown due to the surface plasmon resonance (SPR) [38]. The absorption band of the UV-visible spectrum for the biofabricated AS-AgNPs appeared at 448 nm, which is almost the same absorbance peak as the biosynthesized AgNPs using an extract of *Cymbopogon citratus* [39]. Similarly, a maximum absorbance at 450 nm was observed in the visible UV spectra of biosynthesized silver nanoparticles using an *Acer oblongifolium* extract [40]. Consistent with ref. [41], our study showed that the UV–Vis spectra of green synthesized AgNPs, using the extract of *Allium cepa* var. *Aggregatum*, gives a sharp peak from 410 to 470 nm. Our results are also in agreement with the results of [42], who biofabricated AgNPs using Aloe vera extract, where the UV–Visible spectrum showed a strong broad peak at 455 nm. Generally, AgNPs are known to exhibit a UV–Visible absorption maximum in the range of 400–500 nm because of surface Plasmon resonance [43].

TEM showed the size of AS-AgNPs particles to be about 21.99 nm with a spherical shape. AS-AgNPs with these unique structures have formerly been identified [44]. Additionally, the XRD confirmed the occurrence of AgNPs and their characteristic metallic silver crystalline structure. FTIR is an essential means for the identification of functional groups and interactions between molecules. Therefore, we compared the FTIR spectrum of plant extract with AS-AgNPs. The FTIR spectrum for the biosynthesized AS-AgNPs displayed some shifting in the peaks and changes in peaks intensity. The shifting in the peaks signified that the responsible functional groups were involved in the binding mechanism on the AS-AgNPs. All the peak changes support the role of the functional groups of *A. santolina* extract in reducing and stabilizing agents to biosynthesize AS-AgNPs. It has been demonstrated previously that both the primary and secondary amines are responsible for the stabilization of AgNPs [45]. Our FTIR showed peaks associated with primary and secondary amines capped on the nanoparticle surfaces. These results are consistent with the stated results of bio-fabricated AgNPs using *Urtica diocia* leaves [46]. EDX of the biosynthesized AS-AgNPs showed a characteristic optical absorption band at 3 keV, which established the occurrence of elemental silver in the form of silver nanoparticles [47]. AS-AgNPs were considered very stable in the dispersion medium, depending on the previously described zeta potential for other nanoparticles, which are either higher than +30 mV or lower than −30 mV [48]. Our results revealed that the value of the zeta potential for the *A. Santolina* extract is −80.7 mV. This value reveals the higher stability of *A. Santolina* extracts, in order to biosynthesize AS-AgNPs. The negative value of the zeta potential signifies that the negative surface charge of *A. Santolina* extract could come from the OH−, COO−, CO− functional groups, which are confirmed in FTIR spectrum.

Our results displayed that the MIC of AS-AgNPs was 128 µg/mL. This is the first study to determine the antifungal potential of biosynthesized AgNPs using *A. santolina* extract against the growth of *T. rubrum* in vitro and in vivo. Interestingly, our results confirmed the superior antifungal action of AS-AgNPs over plant extract. This might be due to the fact that AgNPs acted as a carrier of plant phytochemicals, which are used as capping and stabilizing agents, facilitating penetration into fungal cells. Ag^+^ also forms complexes with bases contained in DNA and is a potent inhibitor of fungal DNAases [49]. Furthermore, AgNPs might be able to adhere to fungal hyphae, destroying the fungal cells. Few studies have reported the significant antifungal action of biosynthesized AgNPs using different synthesis methods or different types of extracts against different types of dermatophytes. For example, the results of the serial dilution plate counting method showed that the MIC of chemically synthesized AgNPs against *Microsporum canis*, *Trichophyton mentagrophytes*, and *Microsporum gypseum* is 200, 180 and 170 µg/mL, respectively [50]. The results of the agar diffusion assay revealed a MIC of 30 µg/mL for biosynthesized AgNPs using the extract of the red yeast *Phaffia rhodozyma* against *Microsporum and Trichophyton dermatophytes* [51]. Additionally, the results of the broth microdilution assay revealed that AgNPs mediated by a cold atmospheric-pressure air plasma jet displayed antifungal activity against different dermatophytes with *Epidermophyton floccosum*, which was the most sensitive fungus; meanwhile, *T. rubrum* was the most tolerant, with an MIC ranging from 50 to 100 µg/mL, depending on the fungal species [52]. Furthermore, the results of the agar disc diffusion method showed that AgNPs biosynthesized using the leaf extract of *Passiflora caerulea* displayed antifungal action against *T. mentagrophytes*, *T. rubrum*, *E. floccosum*, *M.audouinii*, and *M.Canis*, of these, *T. rubrum* has an inhibition zone of 104 mm at 75 µg/mL of AgNPs [53]. Moreover, the results of the Oxford cup plate assay revealed that AgNPs synthesized using the cell-free extract of *Lysinibacillus fusiformissp* displayed an antifungal potential against *C. albicans, T. rubrum, and T. mentagrophytes* with an MIC of 30, 50, and 60 µg/mL, respectively [54]. Therefore, our study provides biosynthesized AgNPS using *A. santolina* extract as a novel antifungal drug for the treatment of dermatophyte infection caused by *T. rubrum.*

Interestingly, the extract of *A. santolina* exhibited significant antifungal activity against different fungi, including, *C. albicans, C. tropicalis,* and *C. parapsilosis* because of the occurrence of biologically active antifungal compounds. These include flavonoids, which have nutritional and organoleptic characteristics. Luteolin, 3′,4′,5,7-tetrahydroxyflavone, Rutin, and Apigenin have been reported to show high antioxidant, antibacterial and anti-inflammatory activities and are used for treating hypertension, inflammatory diseases, and cancer [11,55,56].

In this study, AS-AgNPs interrupted mycelial growth, as was demonstrated in the dry mycelium mass examination, which reveals the production of fungal cell material. In dermatophytosis, hyphal production is significant as they penetrate into the deeper layers of the epidermis [57]. Additionally, *T. rubrum* conidia are identified as the primary means of establishing dermatophytosis. They adhere to epithelial cells of the keratinized tissues of the host, germinate and start an invasion of the stratum corneum [1]. Therefore, the results of our study are of specific importance because AS-AgNPs inhibit the conidial germination of *T. rubrum*.

Numerous mechanisms have been described to identify the mode of antifungal activity of AgNPs. These include the ability of AgNPs to destruct the cell membrane permeability barrier and to damage the membrane lipid bilayers, causing the leakage of ions, accompanied by the creation of pores, and decomposing the membrane potential. Additionally, AgNPs might block the cell cycle at the G2/M phase [58]; this results in increasing the production of reactive oxygen species (ROS) and reducing the activity of antioxidant enzymes [59].

The ultrastructural alterations of *T. rubrum* treated with AS-AgNPs include modified mycelial structures, increased cell membrane permeability, and cell wall damage. This is the first report to describe the ultrastructure modifications in *T. rubrum* upon exposure to AgNPs. Other reports have described the morphological and ultrastructural modifications of *T. rubrum* hyphal cells that were treated with different antifungal agents. These include ME1111, which exerted its antifungal action through interfering with active transport system found in the cell membrane with subsequent cell lysis [60], and K101 nail solution, which disrupted the contact between the cell wall and the inner membrane [61].

Interestingly, our data showed the efficiency of AS-AgNPs in producing a 93% leakage of intracellular material, in comparison to the control. Further, the treatment of dermatophytes with 10 µg/mL of AgNPs led to a significant increase in leaked materials, reaching a maximum value of 48% in the case of *M. gypseum* [52]. Additionally, AgNPs might bind to the cytoplasmic materials of damaged cells, leading to the leakage of cytoplasmic constituents and a subsequent cell death.

Ergosterol is a distinctive lipid steroid of the fungal cell membrane that regulates the membrane fluidity, and its biosynthesis pathway is one of the main targets for many antifungal agents [62]. In this study, AS-AgNPs significantly decreased the activity of the CYP51 enzyme, the major enzyme in ergosterol biosynthesis. Ergosterol content reduced dramatically, suggesting the targeting of ergosterol synthesis by AS-AgNPs in a way similar to fluconazole [63]. AgNPs were shown to reduce the ergosterol content in *C. albicans* [64,65]. Additionally, treatment of the *T. rubrum* cells with AS-AgNPs resulted in an increase in the uptake of SYTOX^®^ Green, as a result of the increased membrane permeability of *T. rubrum*. Thus, a reduction in ergosterol after AgNP treatment could be accountable for the sensitivity of fungal cells, the compromised membrane integrity, and changes in the cellular microenvironment. In addition, targeting the fungal cell wall is a favorable mechanism for many antifungal drugs [66]. As an important component of the fungal cell wall, β-1,3-glucan played a significant role in cell growth to confirm the stability of cell osmotic pressure [67]. The suppression of β-(1,3)-d-glucan synthase resulted in the inhibition of the synthesis of glucan and the damage of cell wall [67]. Our results demonstrated the inhibitory effect of AS-AgNPs on the activity of β-(1,3)-d-glucan synthase of *T. rubrum*. In addition, AS-AgNPs suppressed the chitin synthase activity, which is responsible for the production of chitin, an important component of the fungal cell wall that maintains its integrity [68].

AS-AgNPs also elevated ROS production, which consequently resulted in damage to the fungal cells [69]. In this context, Bokyoung et al., 2019, revealed that ROS levels were significantly increased in *C. albicans* in the presence of 50 μg/mL of AgNPs at 30 min [70]. Additionally, in yeast, the toxic action of AgNPs might be due to the generation of ROS via the release of silver ions inside the fungal cells internalizing AgNPs [70].

Infection by *T. rubrum* was shown to stimulate the activation of nuclear factor kappa beta (NF-κB) and the production of IL-1β, IL-6, TNF-alpha and IL-10 by macrophages [71,72,73,74]. Interestingly, the direct inhibitory action of AS-NPs on the fungal burden was associated with the downregulation of pro-inflammatory cytokines, including TNF-α, MOP, IL-1, IL-6, IL-2 and IL-17. Our biosynthesized AS-AgNPs recapitulate the effect of nitric oxide-releasing AgNPs, which have been recently shown to down-regulate the production of IL-2, 6, 10 and TNFα, upon topical application in a mouse model of *T. rubrum* dermatophytosis [75].

Green synthesized AgNPs have several advantages over chemically synthesized AgNPs in topical treatment, including limited cytotoxicity, a low stimulatory effect on the host inflammatory response [76], and the presence of phytochemicals adsorbed on the NP surface, which increases their antimicrobial activity in medical applications [77,78].

To our knowledge, this is the first report to provide the topical use of green biosynthesized AgNPs alone as an effective therapy for *T. rubrum*-induced dermatophytes. Few studies have investigated the anti-dermatophytic effect of chemically synthesized AgNPs in a combination with other agents. For example, nitric oxide-releasing AgNPs have been reported to be effective as topical treatment against *T. rubrum* in a mouse model of dermatophytosis [75]. In addition, a combination of chemically synthesized AgNPs with an atmospheric-pressure air cold plasma jet [52], or with a Nd:YAG laser, was reported to treat deramatophytes in a guinea pig disease model [79]. Our data provide a short treatment course, which lacks the risk of antimicrobial resistance and the systemic side effects of other antifungals. More translational research is needed to determine the antifungal activity of AS-AgNPs against other models of dermatophytosis.

## 5. Conclusions

For the first time, we report the use of an aqueous extract of *A. santolina* as a rapid and sustainable source for the green synthesis of silver nanoparticles. As an alternative therapeutic strategy, we investigated the anti-dermatophytic potential of AS-AgNPs in vitro and in vivo. Our results revealed the significant antifungal action of AS-AgNPs against *T. rubrum* with an MIC of 128 μg/mL. AS-AgNPs led to the inhibition of the conidial germination and hyphal growth, increased the leakage of intracellular material, the inhibition of CYP51, increased the uptake of SYTOX^®^ Green, the inhibition of the activity of β-(1,3)-d-glucan synthase and chitin synthase, the inhibition of ergosterol synthesis, and the complete inhibition of the germination of spores. Topical treatment of dermal *T. rubrum* infection using AS-AgNPs in a rat model displayed a significant improvement in the skin structure repair without showing any in vivo cytotoxicity. The therapeutic potential of AS-AgNPs in vivo was found to be mediated via the inhibitory effect of AS-AgNPs on the fungal burden associated with the downregulation of pro-inflammatory cytokines. Our data provide the information for a short treatment course of dermatophytosis, which lacks the risk of antimicrobial resistance and the systemic side effects of other drugs. The clinical success of AgNPs will pave the way for a new generation of wide-ranging Ag-containing therapeutic products for controlling and preventing the further outbreak of diseases.

## Data Availability

All materials are available by the corresponding author.

## Supplementary Materials

The following supporting information can be downloaded at: https://www.mdpi.com/article/10.3390/molecules28041536/s1, Table S1: Antifungal activity of AS-AgNPs and A. sanotlina extract against T. rubrum.
