# Supplementary material for: Potential Treatment of Dermatophyte Trichophyton rubrum in Rat Model Using Topical Green Biosynthesized Silver Nanoparticles with Achillea santolina Extract
Source: Molecules. 2023 Feb 5;28(4):1536. doi: 10.3390/molecules28041536 (PMC9965404; doi:10.3390/molecules28041536)
Supplement: Supplementary file 1 [file molecules-28-01536-s001.zip › molecules-2174983-supplementary.pdf]

Supplementary Table 1. Antifungal activity of AS-AgNPs and *A. sanotlina* extract against *T. rubrum*

<table>
<tr><th rowspan="3">Concentration
(µg/ml)</th><th colspan="3">Antifungal agent</th></tr>
<tr><th>Terbinafine</th><th>Plant extract</th><th>AS- AgNPs</th></tr>
<tr><th colspan="3">IZD (mm)</th></tr>
<tr><td>0</td><td>$_{a}0^{a}\pm0.0$</td><td>$_{a}0^{a}\pm0.0$</td><td>$_{a}0^{a}\pm0.0$</td></tr>
<tr><td>2</td><td>$_{b}7^{b}\pm0.7$</td><td>$_{b}4^{a}\pm0.7$</td><td>$_{b}10^{c}\pm0.7$</td></tr>
<tr><td>4</td><td>$_{c}9^{b}\pm0.2$</td><td>$_{c}6^{a}\pm0.5$</td><td>$_{c}13^{c}\pm0.5$</td></tr>
<tr><td>8</td><td>$_{d}12^{b}\pm0.4$</td><td>$_{d}9^{a}\pm0.2$</td><td>$_{d}17^{c}\pm0.8$</td></tr>
<tr><td>16</td><td>$_{e}14^{b}\pm0.5$</td><td>$_{e}12^{a}\pm0.5$</td><td>$_{e}21^{c}\pm0.6$</td></tr>
<tr><td>32</td><td>$_{f}19^{b}\pm0.3$</td><td>$_{f}16^{a}\pm0.8$</td><td>$_{f}25^{c}\pm0.3$</td></tr>
<tr><td>64</td><td>$_{g}23^{b}\pm0.5$</td><td>$_{g}19^{a}\pm0.4$</td><td>$_{g}38^{c}\pm0.9$</td></tr>
<tr><td>128</td><td>$_{h}29^{b}\pm0.6$</td><td>$_{h}22^{a}\pm0.8$</td><td rowspan="3">No growth</td></tr>
<tr><td>256</td><td rowspan="2">No growth</td><td>$_{i}25^{a}\pm0.7$</td></tr>
<tr><td>512</td><td>No growth</td></tr>
</table>

IZD= Inhibition zone diameter (mm)

Data are expressed as the mean zone of inhibition in mm followed by SD. The values with different subscript letter in the same column and those with different subscript letter in the same raw are significantly different according to ANOVA and Duncan's multiple range tests.
